# Environment-based object values learned by local network in the striatum tail

**DOI:** 10.1073/pnas.2013623118

**Published:** 2021-01-18

**Authors:** Jun Kunimatsu, Shinya Yamamoto, Kazutaka Maeda, Okihide Hikosaka

**Affiliations:** ^a^Laboratory of Sensorimotor Research, National Eye Institute, NIH, Bethesda, MD 20892;; ^b^Division of Biomedical Science, Faculty of Medicine, University of Tsukuba, 305-8577 Tsukuba, Japan;; ^c^Transborder Medical Research Center, University of Tsukuba, 305-8577 Tsukuba, Japan;; ^d^Integrative Neuroscience, Human Informatics and Interaction Research Institute, National Institute of Advanced Industrial Science and Technology (AIST), 305-8568 Tsukuba, Japan

**Keywords:** striatum tail, local network, scene-environment, saccade, primate

## Abstract

Basal ganglia contribute to object-value learning, which is critical for survival. The underlying neuronal mechanism is the association of each object with its rewarding outcome. However, object values may change in different environments and we then need to choose different objects accordingly. The mechanism of this environment-based value learning is unknown. To address this question, we created an environment-based value task in which the value of each object was reversed depending on the two scene-environments (X and Y). After experiencing this task repeatedly, the monkeys became able to switch the choice of object when the scene-environment changed unexpectedly. When we blocked the inhibitory input from fast-spiking interneurons (FSIs) to medium spiny projection neurons (MSNs) in the striatum tail by locally injecting IEM-1460, the monkeys became unable to learn scene-selective object values. We then studied the mechanism of the FSI-MSN connection. Before and during this learning, FSIs responded to the scenes selectively, but were insensitive to object values. In contrast, MSNs became able to discriminate the objects (i.e., stronger response to good objects), but this occurred clearly in one of the two scenes (X or Y). This was caused by the scene-selective inhibition by FSI. As a whole, MSNs were divided into two groups that were sensitive to object values in scene X or in scene Y. These data indicate that the local network of striatum tail controls the learning of object values that are selective to the scene-environment. This mechanism may support our flexible switching behavior in various environments.

## Significance

Choosing good objects is a fundamental behavior for all animals, to which the basal ganglia (BG) contribute extensively. However, the object choice needs to be changed in different environments. The mechanism of object choice is based on the neuronal circuits originating from output neurons (MSNs) in the striatum. We found that the environment information is provided by fast-spiking interneurons (FSIs) connecting to the MSN circuit. More critically, the experimental reduction of the FSI-input to MSNs disabled the monkey to learn the environment-based object choice. This proved that the object choice controlled by the downstream BG circuit is modulated by the environmental context controlled by the internal circuits in the top of BG circuit. This is important for our flexible decision.

For animals (including humans) to survive, one of the most important behaviors is to obtain valuable objects. It has been shown that the basal ganglia play a crucial role in choosing good objects and rejecting bad objects (1, 2). This basal ganglia mechanism is, at least partially, controlled by dopamine (DA) neurons that encode the values of individual objects (3, 4) and project to the striatum (5, 6). Then, neurons in the basal ganglia change their responses to the objects, thus changing the outputs of the basal ganglia (7–11). This occurs by the gradual change in synaptic weight across the basal ganglia circuit (12–14).

However, this may not be the only mechanism for object choice. The choice often changes based on various contexts, including uncertainty, aversiveness, novelty, and environment (15, 16). How then do the contexts modify the object choice mechanisms? The answer is still unclear. For example, DA neurons changed their responses based on a position-sequence context (17). Cholinergic interneurons in the striatum (tonically active neurons, TANs) responded to visual objects differently depending on the variability of predicted reward (18, 19). These data are very important, but an important question remains: How does the brain encode contexts and control object choice?

We thus decided to study the neuronal mechanism of the context effect. Here, we chose another important context: Environment. In real life, the value of a particular object can be high in one environment but low in the other environment. This context is unique because the brain must be able to reverse the value information of each object suddenly whenever the environment changes. Such “environmental flexibility” is critical for animals (including humans) to survive (20) and is necessary for mental health (21). Here, we tried to reveal the underlying neuronal mechanism.

This raises another question about the neuronal mechanism. As described above, the object-choice mechanism in the basal ganglia is largely based on dopaminergic inputs that are based on the gradual change in synaptic weight (5, 12, 13). However, this mechanism alone would not be able to switch the choice of objects immediately when the environment changes, due to the gradual change in synaptic weight.

Based on a new behavioral procedure, we found that the tail of the striatum (STRt, caudate, and putamen) enables the switching of object choice when the environment changes unexpectedly. This is accomplished by the two groups of neurons: output neurons (medium spiny neurons, MSNs) and inhibitory interneurons (fast-spiking interneurons, FSIs).

## Results

### Monkeys Switch Object Choices Based on the Scene-Environment.

**Fig. 1.**
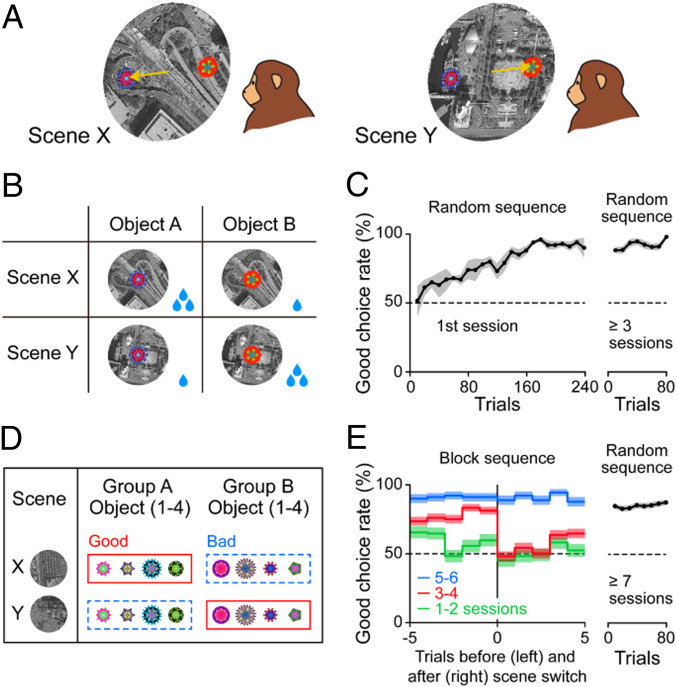
Scene-based object-value task. (*A*) In each trial one of two scenes appeared, and then two objects appeared simultaneously at random positions within the scene. The monkey then chose one of the objects by making a saccade to it. The task procedure is shown in *SI Appendix*, Fig. S1*A*. (*B*) The values of the two objects (objects A and B) switched between the two scenes (X and Y) in opposite manners: A large reward associated with object A in scene X or object B in scene Y (i.e., good); a small reward associated with object A in scene Y or object B in scene X (i.e., bad). (*C*) Learning of good object choice in the first session (*Left*) and the late session (*Right*). Switching of good object choice was often necessary because the two scenes appeared randomly across trials. The data are based on five sets of learning in two monkeys. Even though the late session was done 3 to 10 d after the previous learning, the monkeys were able to do the across-scene switching of good object choice from the beginning of this session. (*D*) The same task, except that eight objects (not two objects) were divided into two groups (groups A and B). In each scene (e.g., scene X), one group (e.g., group A) was associated with a large reward (good) and the other group (e.g., group B) with a small reward (bad). This reward association was reversed in the other scene (e.g., scene Y). In each trial two objects appeared as shown in *A*, one in group A and the other in group B as a random combination. (*E*) Learning of good object choice during the task shown in *D*. During the first 6 sessions, scenes were switched every 20 trials (block sequence, *Left*). This required the switching of the good object group (e.g., any of group A to any of group B) after the scene switch, which is shown in this graph. After the six sessions, scenes were presented randomly across trials (random sequence, *Right*), and the monkeys were able to do the across-scene switching of good object choice from the beginning of this session.

Environment is perceived typically as a large visual scene (22, 23). We thus generated many large visual scenes from satellite imageries (Google Earth). Two scenes are shown in Fig. 1*A*, which were used for the scene-based object-value task. After a scene appeared, two fractal objects appeared at random positions within the scene and the monkey was required to choose one of them by making a saccade from the center dot to the object. A big reward was given if the object was good, but a small reward if the object was bad. If the values of these objects are fixed, the monkeys would learn to choose the good object within several trials (24).

During the scene-based object-value task, however, the learning process became complex (Fig. 1*B* and *SI Appendix*, Fig. S1*A*): Each of the two objects (A and B) can be good or bad, depending on the scene (X or Y). Since the two scenes appear in random sequences, the monkey needed to switch the object choice if the scene has been changed. It took many trials (>160) for the monkey to learn this procedure (Fig. 1 *C*, *Left*). After repeating this procedure a couple of times, however, the monkey was able to switch the object choice, even immediately after the scene changed (Fig. 1 *C*, *Right*).

In another version of this task, eight fractal objects (groups A and B) were presented in each scene (Fig. 1*D*). In each trial, two objects appeared similarly to that shown in Fig. 1*A*, but with a random pair (one of group A and one of group B) in scene X or scene Y. Since this version is more complex, the learning process was done in a block manner for the two scenes (Fig. 1 *E*, *Left*). After three to four sessions of learning, however, the monkey’s choice became significantly good (Fig. 1*E*, red), which became random for several trials in the next block (i.e., after scene change). But after five to six sessions of learning, the monkey was able to switch the choice among many objects (*n* = 8) immediately after the scene change (Fig. 1*E*, blue). Then, the monkey became able to switch the choice of many objects, even when the two scenes appeared randomly (Fig. 1 *E*, *Right*). Such switching behavior was extended to many scenes and objects (*SI Appendix*, Fig. S1*C*). Once the monkeys have learned this extensively, their choice behavior became automatic, since the choice tended to occur even when reward was not delivered after saccades to good objects (*SI Appendix*, *SI Materials and Methods*, and Fig. S2).

### Role of FSIs in the Striatum for Scene-Based Object-Value Learning.

According to our previous research, the basal ganglia play a crucial role in choosing good objects and rejecting bad objects (1, 2). This is done consistently by the population of output neurons (MSNs) in the STRt (caudate and putamen) (9, 11) whose outputs are mediated by the superior colliculus (SC) and are used for gaze bias toward good objects (25). How then can this circuit switch the output based on the environment?

**Fig. 2.**
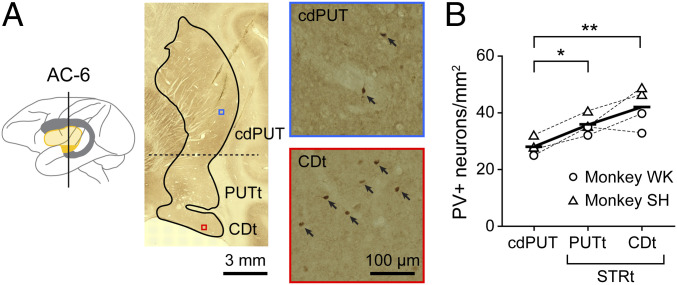
Anatomical and electrophysiological properties of PV^+^ neurons. (*A*) Distribution of PV^+^ neurons in the striatum. (*Left*) Caudate nucleus (gray) and putamen (yellow). AC-6: 6-mm posterior to the anterior commissure. (*Center*) Coronal section of the striatum (AC-6). (*Right*) PV immunostaining sections in cdPUT (top part of panel) and CDt (bottom part of panel). Their locations are shown by small squares (blue and red) in the coronal section (middle of panel). PV^+^ neurons are indicated by arrows. (*B*) Density of PV^+^ neurons in three regions. Differences across regions were assessed using a Tukey–Kramer test (*P < 0.05, **P < 0.01).

Within the striatum there are several groups of interneurons in addition to MSNs (26). Previous neuronal-behavioral studies suggested that FSIs contribute to different aspects of motor performance (27–29). We therefore examined the distributions of FSIs in the tail of caudate (CDt), tail of putamen (PUTt), and caudal-dorsal part of putamen (cdPUT) in monkeys (Fig. 2). FSIs selectively express parvalbumin (PV) (26, 30), which is detected by immunohistochemical staining (*Materials and Methods*). As shown in Fig. 2*A*, many PV^+^ neurons were found in the striatum of monkey WK, especially its ventral part (STRt: CDt and PUTt). PV^+^ neurons were more common in the STRt than in the cdPUT (Fig. 2*B*) (*P* = 0.0012, two-way ANOVA; *P* < 0.01, Tukey–Kramer). This result raised the possibility that the FSIs in the STRt (CDt and PUTt) contribute to the switching of object choice based on the environment.

To test this hypothesis, we blocked the inhibitory inputs from FSIs to MSNs during the learning of scene-based object values. This was done by local injection of IEM-1460, an inhibitor of AMPA receptors lacking the GluA2 subunit. This drug selectively blocks synaptic excitation of FSIs but not MSNs or other interneurons (31, 32).

We first tested the effect of IEM-1460 by recording activity of single FSIs before and after the injection using an injectrode (*SI Appendix*, Fig. S3). FSIs were tonically active, but their firing rates decreased quickly after IEM-1460 injection (*SI Appendix*, Fig. S3), which continued over 120 min. Moreover, their selective responses to scenes disappeared, which is shown for one FSI (*SI Appendix*, Fig. S3*A*) (*z* = 4.49, *P* = 7.21 × 10^−6^; two-sided Wilcoxon rank-sum test) as well as all recorded FSIs (*P* < 0.05, *n* = 6) (*SI Appendix*, Fig. S3*B*), but not after saline injection (*P* = 0.18, *n* = 1). These results establish that the scene-selective signals from FSIs are blocked by IEM-1460.

**Fig. 3.**
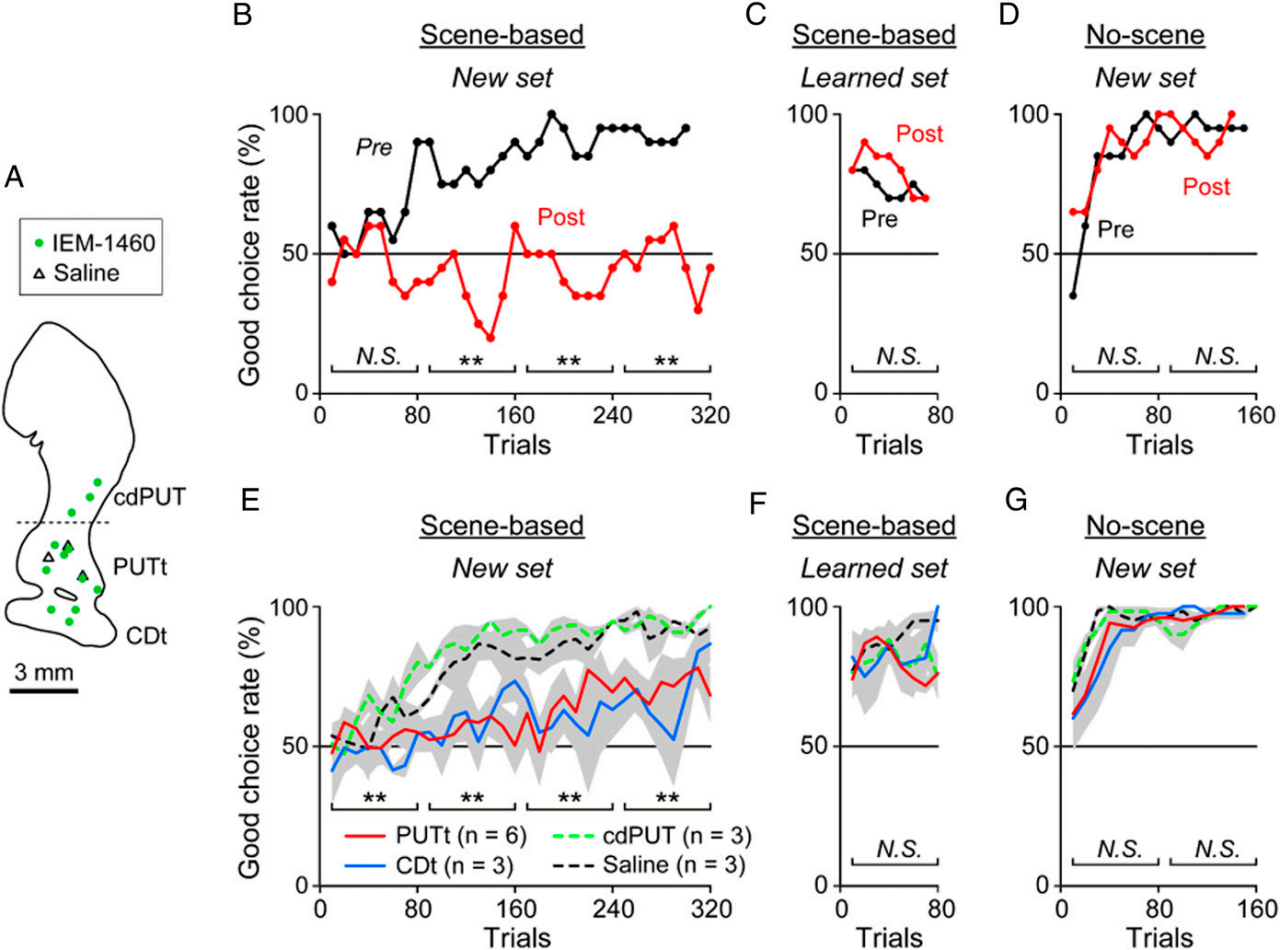
Lack of learning when FSIs are blocked. (*A*) Locations of IEM-1460 and saline injections. (*B*–*D*) Example data on object value learning. (*B* and *C*) Effect of IEM-1460 on the choice of good objects during the scene-based object-value task (Fig. 1*B*) with two objects that were new (*B*) or already-learned (*C*). (*B*) With a pair of new objects, the choice rate of good object increased gradually before IEM-1460 injection, but this learning disappeared after the IEM-1460 injection (**P < 0.01, χ^2^ test). (*C*) With a pair of already-learned objects, the choice rate of good object was high from the beginning before IEM-1460 injection. This already learned behavior was not affected after IEM-1460 injection (*P* = 0.94, χ^2^ test). (*D*) The choice of good objects during the no-scene object-value task with eight new objects (no scene, no switching) was not affected by IEM-1460 injection in both early and late stages (*P* > 0.43, χ^2^ test). (*E*–*G*) Population data corresponding to *B*–*D*. (*E* and *F*) Averaged choice rate of good object shown separately for four conditions. The deficit of new object learning (*E*), not already-learned objects (*F*), occurred selectively by IEM-1460 injection in PUTt and CDt. This was very different from the other conditions (IEM-1460 injection in cdPUT and saline injection) throughout the learning stages (**P < 0.01, two-sided paired *t* test). (*G*) No effect of IEM-1460 on the no-scene object-value task in both early and late stages (*P* > 0.07, two-sided paired *t* test). N.S., not significant.

We then injected IEM-1460 separately in the CDt, PUTt, and cdPUT (Fig. 3*A*), and examined the monkey’s performance in three procedures: 1) Scene-based object-value task with new objects (Fig. 3 *B* and *E*), 2) scene-based object-value task with well-learned objects (Fig. 3 *C* and *F*), and 3) no-scene object-value task with new objects (Fig. 3 *D* and *G*). The scene-based object-value task is the same as the one shown in Fig. 1 *A* and *B*, except that the two objects were presented in the same hemisphere (i.e., contralateral or ipsilateral to the IEM-1460 injection site). In the no-scene object-value task, eight objects appeared with no scene that were divided into good and bad objects consistently (nonswitching) (*SI Appendix*, Fig. S4*A*). Each procedure was tested before and after the injection of either IEM-1460 or saline at one location in the striatum.

Fig. 3*B* shows the results before and after the injection of IEM-1460 in the PUTt. Before the IEM-1460 injection, the monkey steadily learned the scene-based value switching of new objects over the course of 200 trials (Fig. 3*D*, Pre). After the IEM-1460 injection, however, there was virtually no learning, even after 300 trials (Fig. 3*B*, Post). Monkeys did switch the object choice, but not following the scene change. This deficit occurred when the objects were presented either contralateral or ipsilateral hemifield. The data suggest that FSIs are necessary to enable the scene-selective object choice. Similar effects occurred repeatedly by the injection of IEM-1460 in the STRt (PUTt and CDt), although there was slight learning on average (Fig. 3*E*). In contrast, there was no learning deficit by IEM-1460 in the cdPUT (Fig. 3*E*). Saline injections in the STRt had no effect. Since each FSI projects to a close-local region of the striatum (33–35), these data suggest that a selective group of MSNs, which are located in the STRt (PUTt and CDt), contribute to the scene-selective object choice.

However, IEM-1460 became ineffective after the learning of the scene-based object-value task was completed. When the same task was tested several days later (3 to 10 d) using the same (learned) objects, the monkeys performed the task significantly well (*P* < 0.05, two-sided paired *t* test) before IEM-1460 was injected (Fig. 3*C*, Pre). In this condition, the monkey’s choice of good objects, which switches across scenes, was largely automatic (*SI Appendix*, Fig. S2). Importantly, such learned performance was unaffected by the injection of IEM-1460 in either the PUTt, CDt, or cdPUT (Fig. 3*C*, Post, and three lines in Fig. 3*F*) (*P* > 0.05), unlike the initial learning (Fig. 3 *B* and *E*). Second, IEM-1460 did not affect the learning of the no-scene object-value task with new objects (Fig. 3 *D* and *G*) (*P* > 0.05) in which the value of each object did not switch (as shown in *SI Appendix*, Fig. S4). This is different from the effect of the muscimol injection in the CDt (i.e., inactivation of MSNs and others), which disabled the discrimination of good and bad objects (8). These results suggest that FSIs are necessary for the learning of good objects, which switches across scenes, but become unnecessary after the automatic choice has been acquired.

### MSNs Encode Scene-Based Value Information.

What is the neuronal mechanism of FSIs for scene-based object-value learning? We next examined the responses of MSNs in the STRt, because FSIs have direct inhibitory connections to MSNs and regulate their activity (36, 37). First, we examined one MSN after two types of learning: 1) Without a scene and 2) with two scenes.

During the no-scene learning (no-scene object-value task) (*SI Appendix*, Fig. S4*A*), eight fractal objects were presented randomly and sequentially, half (*n* = 4) always associated with a big reward (good objects) and the other half (*n* = 4) always associated with a small reward (bad objects). After several sessions of learning, the object-value coding was tested using the scene(−)-passive viewing task while the monkey fixed its gaze at the center (*SI Appendix*, Fig. S4*B*). The MSN responded to these objects somewhat differently. Two good objects caused strong response, even though each object was no longer associated with a particular reward. This response pattern is common among MSNs in the STRt (CDt and PUTt), which has been called “stable value-coding” (2, 8, 11). In contrast, MSNs in the anterior part of the striatum (CDh) are selectively sensitive to the upcoming reward and therefore do not respond to objects in the scene(−)-passive viewing task, which has been called “flexible value-coding” (8).

During the two-scene learning (scene-based object-value task) (Fig. 1 *D* and *E* and *SI Appendix*, Fig. S1*A*), half of the objects (*n* = 4, called object A) were always associated with a big reward in one scene (scene X), but with a small reward in the other scene (scene Y). In contrast, the other half (object B) were always associated with a small reward in one scene (scene X), but with a large reward in the other scene (scene Y). After several sessions of learning, we examined the same MSN using the scene(+)-passive viewing task (*SI Appendix*, Fig. S1*B*). In this task the scene appeared at first, and then objects were presented one at a time at the receptive field for the recorded neuron while the monkey fixed its gaze at the center of the scene. The scenes and objects were chosen randomly across trials from a set of two scenes and eight objects, respectively (e.g., Fig. 1*D*). We used multiple objects in order to minimize value-irrelevant object selectivity.

**Fig. 4.**
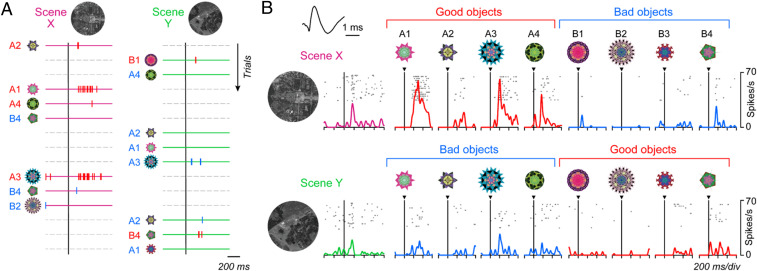
Different responses of MSN to objects depending on their values and scenes. (*A*) Activity of a representative MSN during the scene(+)-passive viewing task (*SI Appendix*, Fig. S1*B*), during which eight objects (A1 to A4, B1 to B4) were presented randomly (top to bottom) in scene X (*Left*) or scene Y (*Right*). Each spike is shown by a vertical bar, whose color indicates good (red) or bad (blue) object; the same color is also shown in the object number. Horizontal bars indicate the different scenes (magenta or green). In this task reward was unrelated to object or scene. (*B*) Average responses of the representative MSN (shown in *A*) to scenes and objects. Data in each combination of rasters and spike density are aligned on the onset of scene (*Left*) and object (*Right*). This is one of the scene X-preferring MSNs. *Inset* shows the waveforms of this neuron’s spike.

Fig. 4*A* shows the activity of the same MSN during scene(+)-passive viewing task. The MSN was excited by two objects (A1, A3) when they appeared in scene X (where they were good), but not when they appeared in scene Y (where they were bad). For example, A3 appeared repeatedly (first in scene Y, second in scene X), but the response was clear only in the second trial. Thus, the MSN changed its response to the same object suddenly based on scene (= environment). In contrast, the MSN showed little response to the other six objects. Such object-selectivity is common among MSNs in the CDt/PUTt (11, 38).

Overall, this MSN responded more strongly in scene X (Fig. 4 *B*, *Upper*) than in scene Y (Fig. 4 *B*, *Lower*) to objects (on average across eight objects, *z* = 2.48, *P* = 0.01; two-sided Wilcoxon rank-sum test), which we call scene X-preferring MSN. In scene X, the responses to good objects were stronger than to bad objects (*z* = 6.31, *P* = 2.77 × 10^−10^), which was similar to the scene(−) condition (*SI Appendix*, Fig. S4). In scene Y, the value-coding became smaller, somewhat stronger to bad objects than good objects (*z* = −2.70, *P* = 0.0069). This occurred mainly because the strong responses to good objects in scene X became much smaller in scene Y (which were now bad objects) (*z* = 4.27, *P* = 3.08 × 10^−3^). In addition, the MSN responded also to these scenes: More strongly to scene X than scene Y (*z* = 2.27, *P* = 0.023; two-sided Wilcoxon rank-sum test) (Fig. 2*B*).

**Fig. 5.**
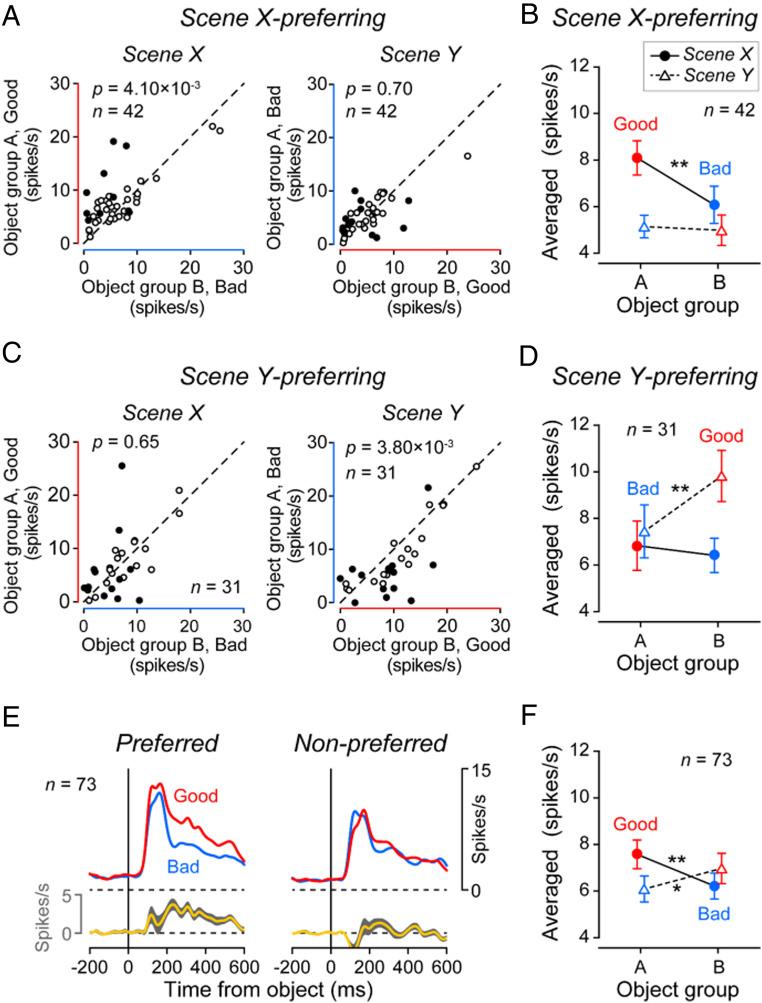
Scene-based value-coding of MSNs. (*A*) Responses of each scene X-preferring MSN to group A objects (ordinate) and group B objects (abscissa) in scene X (*Left*) and scene Y (*Right*). Significantly different responses to group A objects and group B objects are shown by filled symbols (Wilcoxon ranksum test, *P* < 0.05). (*B*) Averaged responses of scene X-preferring MSNs (*n* = 43) to group A objects (left side) and group B objects (right side), shown separately for scene X (circle) and scene Y (triangle). In *A* and *B*, good and bad objects are indicated by red and blue symbols (line, circle, triangle), respectively. Significant value-coding is indicated by asterisk (*P < 0.05, **P < 0.01, two-sided paired *t* test). (*C* and *D*) Responses of scene Y-preferring MSNs (*n* = 30), shown in the same way as in *A* and *B*. (*E*) Time courses of averaged responses of all MSNs (two groups combined, *n* = 73) to good objects (red) and bad objects (blue), which are shown separately for two conditions: when the objects were presented in the preferred scene (*Left*) vs. nonpreferred scene (*Right*). (*F*) Averaged response of all MSNs (two groups combined) to object groups A and B.

To quantify the value coding for MSNs, we divided them into two groups (Fig. 5): Scene X-preferring (*n* = 42) and scene Y-preferring (*n* = 31). Fig. 5*A* shows the responses of individual MSNs in the scene X-preferring group to good vs. bad objects, separately for scenes X and Y. These MSNs, as a whole, responded to good objects strongly in the scene X [*t*(41) = 3.04, *P* = 4.10 × 10^−3^; two-sided paired *t* test] (Fig. 5*B* and *SI Appendix*, Fig. S5 *A*, *Left*), but not in scene Y [*t*(41) = 0.39, *P* = 0.70]. Fig. 5 *C* and *D* show the equivalent data of the scene Y-preferring MSNs. They encoded positive value (good > bad) in scene Y [*t*(30) = −3.14, *P* = 3.80 × 10^−3^], but not in scene X [*t*(30) = 0.46, *P* = 0.65] (Fig. 5*D* and *SI Appendix*, Fig. S5 *A*, *Right*).

Notably, individual MSNs showed the value-coding in the preferred scene, but selectively to individual objects (*SI Appendix*, Fig. S6), as represented in Fig. 4. This is due to the high object-selectively of MSNs (11, 38). However, the combination of the selective value-coding across many MSNs led to the general value-coding of all objects (*SI Appendix*, Fig. S6, Population). The time courses of the population activity for the 73 MSNs again confirmed that MSNs as a whole responded to good objects more strongly than bad objects in the preferred scene (Fig. 5*E*).

MSNs are the final output neurons of the striatum (5); thus, it is likely that the downstream areas in the basal ganglia receive combined information from the two groups of MSNs (Fig. 5 *B* and *D*). The averaged output of all MSNs (Fig. 5*F*) indicates that the value coding of the total STRt output reverses when the scene changes. These signals, which are conveyed to downstream regions (substantia nigra pars reticulata [SNr] or globus pallidus externus [GPe]) (24), may contribute to the scene-dependent behavioral switching. In fact, virtually all caudal-dorsal-lateral SNr (cdlSNr) neurons show stable value-coding of many objects (e.g., >200) (10).

We also tested more pairs of scenes and more groups of objects (*SI Appendix*, Fig. S1*C*). MSNs, as a population, responded to these groups of objects differently across each set of scenes (*SI Appendix*, Fig. S7*A*), although each MSN tended to show clear value discrimination in one of the pairs of scenes (*SI Appendix*, Fig. S7*B*). These data suggest that the combined output of MSNs would enable the choice of good objects in many scenes.

### FSIs Encode Scene Information.

The results so far suggest that MSNs in the STRt encode object values consistently if there is no scene (e.g., *SI Appendix*, Fig. S4). However, when the scene appears to reverse the object values, the value coding is suppressed in nonpreferred scenes (e.g., Fig. 4). This result suggested that MSNs are inhibited by selective scenes (e.g., scene Y), which suppressed object value-coding.

**Fig. 6.**
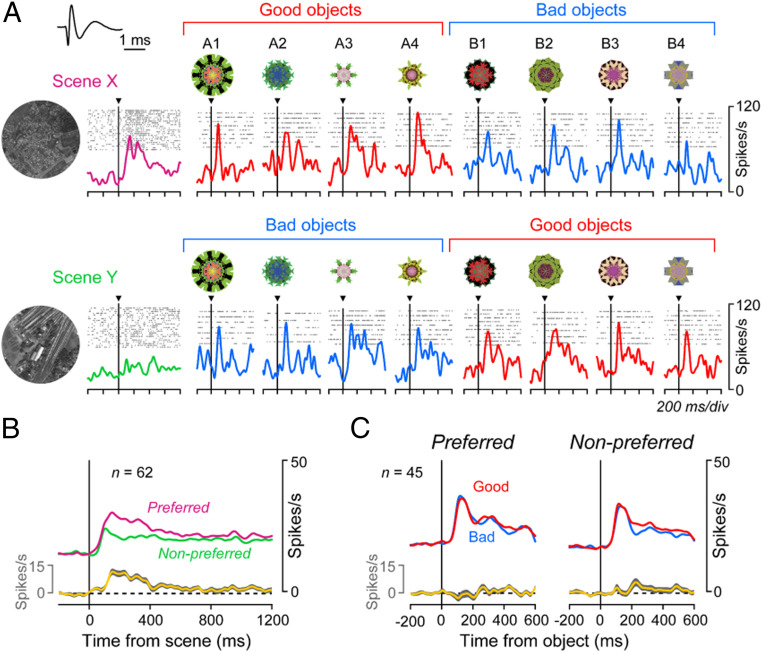
Scene-selective responses of FSIs. (*A*) Activity of a representative FSI during the scene(+)-passive viewing task in scene X (*Upper*) and scene Y (*Lower*). *Inset* shows the waveforms of this FSI. (*B*) Time course of averaged responses of FSIs to the preferred scene (magenta) and the nonpreferred scene (green). The yellow line indicates the difference between the preferred and nonpreferred responses (mean ± SE). Equivalent data for individual FSIs are shown in *SI Appendix*, Fig. S10*B*. (*C*) Averaged responses of FSIs to good objects (red) and bad objects (blue) in the same form as in Fig. 5*E* for MSNs.

We next examined the activity of FSIs during the scene(+)-passive viewing task. FSIs were discriminated from MSNs (*SI Appendix*, Fig. S8) using previously established criteria: Shorter spike duration and higher spontaneous firing rate (27–29, 39, 40). We recorded 236 neurons in the STRt (*SI Appendix*, Fig. S9) and classified 82 FSIs and 115 MSNs. Fig. 6*A* shows the activity of a representative FSI during the passive viewing task. This FSI was excited by scenes, but differently between the two scenes (*z* = 4.18, *P* = 2.91 × 10^−5^, two-sided Wilcoxon rank-sum test). It also responded to objects but showed no significant value-bias (*z* = 0.89, *P* = 0.37).

As a population, many FSIs (62 of 82, 76%) responded to several scenes and did so selectively with different magnitudes (Fig. 6*B* and *SI Appendix*, Fig. S10 *A and B*). For some FSIs, we also tested the scene response by the set that has the same objects (*SI Appendix*, Fig. S1*C*) and found that FSIs responded to scenes selectively regardless of the contained objects (*SI Appendix*, Fig. S7*B*). These data, together with the anatomical data (Fig. 2), suggests that MSNs are inhibited strongly in one of the two scenes (X or Y), which creates two groups of MSNs (scene Y-preferring, scene X-preferring), as shown in Fig. 5 and *SI Appendix*, Figs. S5 and S6. These FSIs also responded to good and bad objects, but nondifferentially both in the preferred or nonpreferred scenes (Fig. 6*C*).

To examine these hypotheses, we collected equivalent data for MSNs (*SI Appendix*, Fig. S10 *C* and *D*). Similarly to FSIs, some MSNs (33 of 115, 29%) responded to several scenes selectively (e.g., Fig. 4*B* and *SI Appendix*, Fig. S10*D*). These excitatory responses are unlikely to be caused directly by the inhibitory inputs from FSIs. Instead, MSNs as well as FSIs may receive excitatory inputs from scene-sensitive brain areas, including the parahippocampal cortex (23, 41). Although many MSNs were not excited by any scene, they may have received such excitatory scene inputs which, however, may not generate action potentials because MSNs are highly hyperpolarized normally (37, 42).

### Learning Effects in MSNs and FSI.

The above results suggest that FSI contributes to the learning of scene-based object values in MSN by inhibiting the MSN with a selective scene, not selective objects. However, the underlying mechanism is unclear because the data so far (Figs. 4 and 5) indicate the final outcome of scene-based object-value learning.

**Fig. 7.**
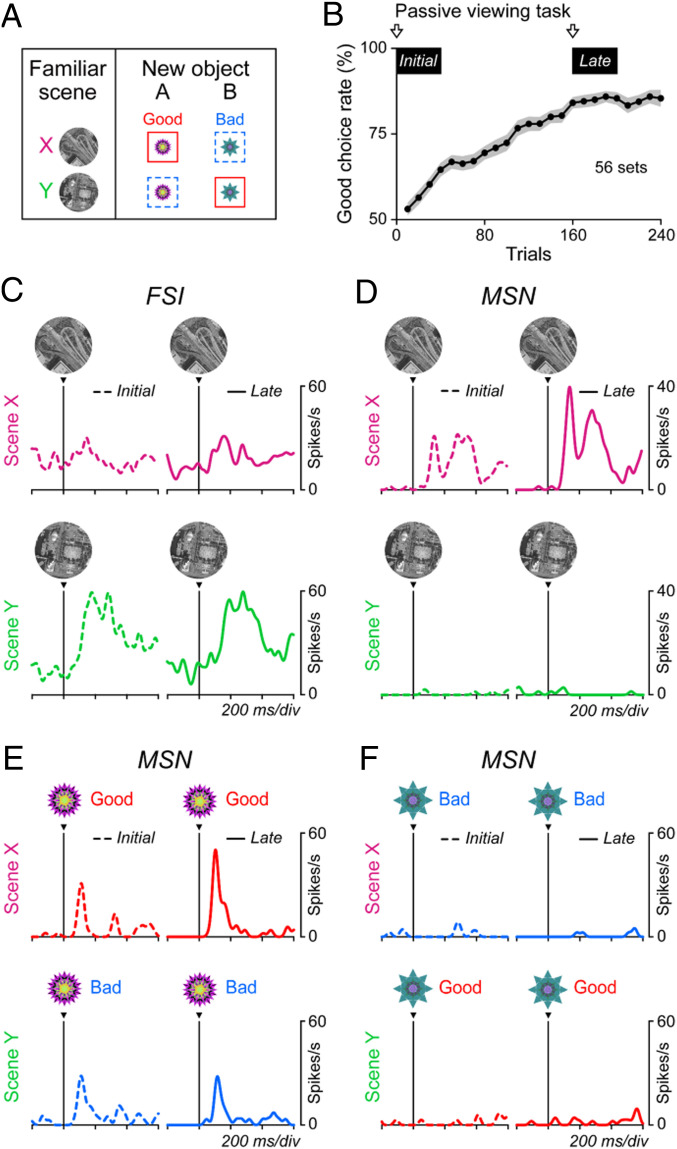
Learning effects in MSNs but not FSIs. (*A*) Scene-based object-value task with two new objects. Same as shown in Fig. 1 *A* and *B* and *SI Appendix*, Fig. S1*A*. (*B*) Learning curve averaged across 56 sets (which were used for neuronal recording) in 2 monkeys. In the initial and late learning phases, we let the monkey perform the scene(+)-passive viewing task to check the responses of MSNs to individual objects. (*C* and *D*) Responses of representative FSI (*C*) and MSN (*D*) to the two scenes (*Upper*: X, *Lower*: Y) during the scene-based object-value task (*A*). *Left* and *Right* indicate the before (dash lines) and after (solid lines) learning during the scene-based object-value learning (*A*), respectively. (*E* and *F*) Responses of the representative MSN to each object during the scene(+)-passive viewing task.

To address this question, we recorded from FSIs and MSNs while the monkeys performed the same scene-based object-value task (Fig. 1*B*). Here, we used two familiar scenes and two new objects (instead of eight objects) (Fig. 7*A*) so that the monkey could learn scene-based values moderately within one session. To check the effect of learning on object responses in MSNs, we also used the scene(+)-passive viewing task (*SI Appendix*, Fig. S1*B*) prior to learning (Fig. 7*B*, Initial) and again late in the learning session (Fig. 7*B*, Late).

The activity of example neurons (one FSI and two MSNs) is shown in Fig. 7 *C*–*F*. Before learning, both FSI and MSN responded to the two scenes differently (Fig. 7 *C* and *D*). MSN responded to the two objects differently (Fig. 7 *E* and *F*), but these responses were not different across the two scenes before learning (Fig. 7 *E* and *F*, *Left*). After 160 trials of learning, the monkey learned the scene-based values of the objects (Fig. 7*B*). This learning was associated with two changes of MSN activity: 1) Response to object A increased in scene X (*z* = −2.11, *P* = 0.03, two-sided Wilcoxon rank-sum test) but not scene Y (*z* = 0.61, *P* = 0.54) (Fig. 7*E*); 2) response to scene X increased (*z* = −2.12, *P* = 0.03), but not scene Y (*z* = −0.03, *P* = 0.98) (Fig. 7*D*). The significant changes of response to scene and object were found in 17% (4 of 24) and 33% (8 of 24) of recorded MSNs, respectively (*P* < 0.05). In contrast, FSI did not change its response to either scene (Fig. 7*C*) (scene X, *z* = −0.61, *P* = 0.54; scene Y, *z* = 1.33, *P* = 0.18). The outcome of the learning in this MSN is similar to another MSN shown in Fig. 4.

These data suggest that the inhibitory inputs from FSIs to MSNs are stable but selective for scenes and consequently the responses of MSNs to both scenes and objects are modulated by the scene-selective inhibition from FSIs.

## Discussion

Our results revealed that the scene-based object value coding of MSNs is suppressed by the inhibitory inputs from the scene-selective FSIs, specifically during learning. The learning process is critical because, after the learning, the switching of object choice across the scenes occurred stably (Fig. 1 *C*, *Right* and Fig. 1 *E*, *Right*) and automatically even a month later (*SI Appendix*, Fig. S2). What then is the neuronal mechanism of the learning? Specifically, how does the neuronal circuit comprising FSIs and MSNs create the scene-based value signal?

**Fig. 8.**
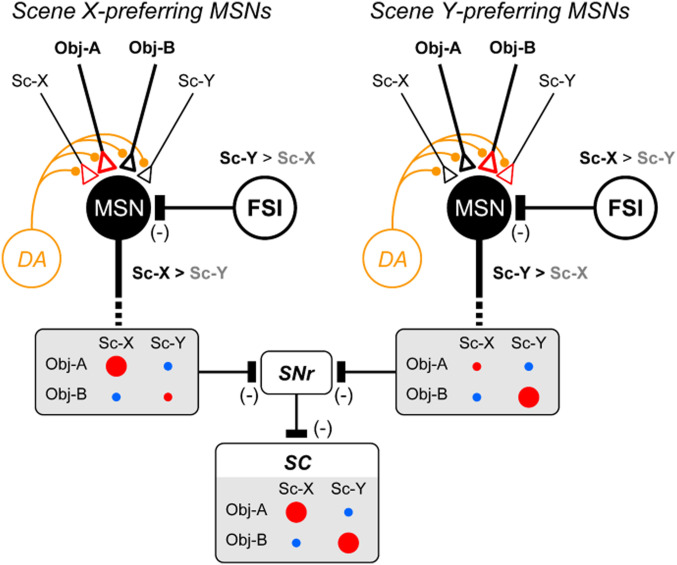
Hypothetical diagram of scene-based object-value learning. MSNs receive excitatory inputs of various objects which are modified by reward-related DA input and scene-related FSI input (as shown in *SI Appendix*, Fig. S11). Inhibitory input from scene Y-preferring FSI generates scene X-preferring MSN (*Left*). Then, its response increases to object A in scene X, but not other conditions (object A in scene Y, object B in scene X, object B in scene Y) (*SI Appendix*, Fig. S11*D*), which is shown by circle size (red: rewarded, blue: nonrewarded). This is caused by two factors during learning: 1) Larger output in scene X (by FSI input), 2) increase in synaptic weight of object A input (red triangle). In contrast, inhibitory input from scene X-preferring FSI creates scene Y-preferring MSN (*Right*), whose response increases to object B in scene Y, but not other conditions. These two groups of MSNs together would generate disinhibitory outputs to the SC neurons through SNr neurons (MSN-SNr inhibition, SNr-SC inhibition). Then, the output of SC neurons is increased (i.e., disinhibited) in two conditions (object A in scene X, object B in scene Y), facilitating saccades to good objects (not to bad objects). In addition, we speculate the scene-selective excitatory input to MSNs. This is effective because the particular combination of object and scene (e.g., object A and scene X) would facilitate the synaptic weight in these inputs together (red triangle). Moreover, such selective scene inputs will keep the learning effect even when the FSI input is removed (Fig. 3 *C* and *F*).

Fig. 8 shows a hypothetical network diagram for scene-object learning. Our data suggest that FSIs are excited by many scenes widely but selectively (Fig. 6 *A* and *B*). Anatomically, FSIs have inhibitory connections onto many MSNs (33, 35). According to the diagram, each MSN is inhibited by several scenes differently through the inhibitory connection from FSI. When scenes X and Y are used, MSNs are inhibited differently by FSIs, which generated two groups: Scene X-preferring MSNs (Fig. 8, *Left*) and scene Y-preferring MSNs (Fig. 8, *Right*).

Importantly, the scene-based object-value learning occurred differently in these two groups of MSNs (Fig. 5). Its basic mechanism is shown in *SI Appendix*, Fig. S11, which is based on three factors (*SI Appendix*, Fig. S11*A*): 1) Excitatory input of each object to MSN mostly from the inferotemporal cortex (43–46) where neurons encode visual object information (47–49), including scene-related signals (50, 51); 2) input from DA neurons (12–14); and 3) inhibitory input of each scene to MSN from FSI (36, 37). The object input is enhanced or depressed if it is followed by a big reward or small reward, respectively, based on DA input. If there is no input from FSI, the object input does not change virtually because the object is associated with a big reward or small reward across the two scenes (e.g., object A in scenes X and Y) (*SI Appendix*, Fig. S11 *C*, *Left*). The final effect after experiencing both scene X and scene Y is shown by a black arrow with a yellow circle in *SI Appendix*, Fig. S11*C*.

However, this is modulated by the inhibitory input from FSI in two ways. First, the response of MSN to either object (A or B) is reduced in the nonpreferred scene (scene Y) (*SI Appendix*, Fig. S11 *D*, *Lower*). Second, the effect of DA input is also modulated by the response of MSN to the object (e.g., weaker if output is lower) (52). For example, scene X-preferring MSN shows a weaker change in object input in scene Y: Lower decrease of object A input (*SI Appendix*, Fig. S11 *D*, *Lower Left*) and lower increase of object B input (*SI Appendix*, Fig. S11 *D*, *Lower Right*). These effects together lead to the selective changes of the object response: higher to object A in scene X (*SI Appendix*, Fig. S11 *D*, *Upper Left*), lower to object A in scene Y (*SI Appendix*, Fig. S11 *D*, *Lower Left*), object B in scene X (*SI Appendix*, Fig. S11 *D*, *Upper Right*), object B in scene Y (*SI Appendix*, Fig. S11 *D*, *Lower Right*). Each effect is shown by a black arrow with yellow circle in each graph. Following the same mechanism, scene Y-preferring MSN would show the higher response to object B in scene Y and lower responses in the other three conditions.

These hypothetical results are illustrated in Fig. 8, which indicates that each MSN responds to the good objects strongly (with reward) and does so selectively in the preferred scene. This is actually what we found experimentally (Fig. 5 *B* and *D*). The model data in *SI Appendix*, Fig. S11*D*, however, show that there are some differences in the other three conditions, which was unclear in the experimental data (Fig. 5 *B* and *D*). This may be because MSNs are highly hyperpolarized normally (37, 42) and therefore unable to generate action potentials often under these insufficient conditions (bad object or nonpreferred scene).

Another important mechanism is the functional integration of the two groups of MSNs. This is done by the inhibitory circuits from both groups of MSNs to single neurons in the SNr, which then inhibits saccadic neurons in the SC (Fig. 8). SC neurons would then be excited by either object (A or B) when it predicts a reward (scene X or scene Y). These hypothetical data indeed correspond to the experimental data of MSNs: Combined responses of the two groups of MSNs (Fig. 5*F*). This also corresponds to the behavioral data: Frequent saccades to good objects (object A in scene X, object B in scene Y) (*SI Appendix*, Fig. S2).

In addition to objects, MSNs often responded (excited) to scenes (Fig. 4*B* and *SI Appendix*, Fig. S10*B*), which is added as excitatory input to MSNs in Fig. 8. Reward is available in two conditions: Object A in scene X, object B in scene Y. For scene X-preferring MSNs, however, the reward effect (by DA input) is weak in the second condition (object B in scene Y) because its output is reduced. Then, the synaptic weight would be increased for the two inputs (object A, scene X) (Fig. 8, *Left*), which is consistent with data in Fig. 7 *D*–*F*. The opposite effect occurs in scene Y-preferring MSNs (Fig. 8, *Right*). These descriptions are what our model has revealed (*SI Appendix*, Fig. S12) and are consistent with our experimental data (Figs. 4, 5, and 7 and *SI Appendix*, Figs. S5 and S6).

Importantly, the scene input to MSNs can explain another experimental effect: After the complete learning, the scene-based object choice was not affected by the blockade of FSI input by IEM-1460 (Fig. 3 *C* and *F*). If the MSN has no scene input and if FSI input is blocked, the MSN would not be able to discriminate the four conditions (object A in scene X, object A in scene Y, object B in scene X, object B in scene Y). Therefore, FSI input is critical for learning of scene-based object choice, but after learning MSN alone can choose objects depending on the scene. This will continue for a long time (e.g., >1 y) because their mechanisms remain stable (i.e., long-term memory) (8, 9).

The STRt circuit fits this function because it uses long-term object-value memories (8, 9, 11) that are controlled by sustain-type DA neurons that are located in the caudal-dorsal-lateral part of the substantia nigra compacta (cdlSNc) (4). Consistent with the needs of long-term memory, neurons along the STRt circuit encode historical values of many objects. Based on our experimental data and model, we propose a new concept: “flexibility based on stability.” When the reward outcome is uncertain or stochastic, neurons in the anterior part of the basal ganglia become very active (53, 54), together with surrounding areas (55), and their inactivation disrupts the early learning of new behaviors (56). Therefore, the anterior basal ganglia contribute to purely flexible behaviors. However, different behaviors are often required in different environments or contexts (16): The behavior needs to be switched flexibly when the environment/context has changed, while each behavior must be set stably in each environment/context. This corresponds to the behavioral procedure we used here. Therefore, flexibility based on stability is a very common type of behavior, but has not been studied extensively for the underlying neuronal mechanism.

Finally, we will describe several speculative ideas that may be useful for future research. A reduction in FSI has been reported in Tourette syndrome (57) and Huntington’s disease (58), which show a dysfunction in skill learning in addition to the motor deficit (59, 60). Recently, some rodent studies revealed that dysfunction of FSI leads to the deficit of associative learning by modulating the activity of MSNs (29, 61). On the other hand, a basic mechanism of learning is the change in the synaptic weight of the direct cortical inputs to MSNs, which is caused mainly by DA (12–14). These findings raise a question: How do these mechanisms interact with each other? Our data provide one answer: DA neurons control learning of object choice, while FSIs select environmental context (i.e., scene) that is necessary for the learning. This function may not be restricted to FSIs. It has been shown that other interneurons in the striatum, especially cholinergic interneurons (TANs), modify learning based on the context (39). Notably, TANs seem to be sensitive to different kinds of context (18). In conclusion, context-dependent learning is important in real life because the goal may change in various contexts, to which FSIs and other interneurons in the basal ganglia contribute selectively and differently.

## Materials and Methods

### Animal Model.

Three adult male rhesus monkeys (*Macaca mulatta*, 8 to 11 kg, 6 to 10 y old) were used for the experiments (monkey WK and monkey SP for the physiological experiment and monkey WK and monkey SH for the histological experiment). All procedures for animal care and experimentation were approved by the Animal Care and Use Committee of the National Eye Institute and complied with the Public Health Service Policy on the humane care and use of laboratory animals. A plastic head holder, eye coil, and plastic recording chamber were implanted under general anesthesia and sterile surgical conditions. After the monkeys fully recovered from surgery, we started training them with the oculomotor tasks.

### Behavioral Procedure.

Behavioral procedure was controlled by C++-based real-time experimentation data-acquisition system (Blip: available at http://www.robilis.com/blip/). The monkey sat in a primate chair, facing a frontoparallel screen in a sound-attenuated and electrically shielded room. Visual stimuli generated by an active-matrix liquid-crystal display projector (PJ550, ViewSonic) were rear projected on the screen. We presented the scene (gray scale, radius: 25°) and object (multiple colors, radius: 5°) as visual stimuli. The scenes were created by using Google Earth imagery (https://www.google.com/earth/) and fractals were made by fractal geometry (38).

### Scene-Based Object-Value Task.

The purpose of this task was to let the subject learn the scene-object association. One of two scene images was presented for 800 ms. After the monkeys had fixated on the red-square fixation point for 600 to 1,000 ms, the fixation cue disappeared and two objects (different value objects) appeared simultaneously in a different hemifield (for training and neuronal test) or same hemifield (for pharmacological experiment). A reward was given after the monkeys make a saccade to the stimulus and maintained fixation for 200 ms. Half of the fractal objects was associated with a large reward (0.3 mL; i.e., good objects), and the other half were associated with a small reward (0.1 mL; i.e., bad objects). We defined the objects associated with a small reward as bad objects even the reward is still positive because monkeys preferred not to choose these objects relative to the objects associated with a large reward (good objects). This reward association changed depending on the scene. For testing the long-term effect (Figs. 4–6), each session was performed with a set of eight new objects and two new scenes. In the initial learning phase, the scene was changed randomly in every 20 trials. We presented the scene randomly after the monkeys started switching their choices when the scene was changed (usually after seven learning sessions). One learning session consisted of 160 trials (8 blocks). Each set was learned in 1 d within a single learning session. For testing the learning effect (Figs. 2 and 7), each session was performed with a set of two new objects and two familiar scenes. In this case, we presented the scene randomly in every trial during all learning sessions.

### Passive Viewing Task.

The purpose of this task was to test for value-biased behavioral responses for learned scene-object combinations. In this task, one of two scene images was presented for 800 ms randomly (*SI Appendix*, Fig. S1*B*). If the monkey fixated on a central red square, two to four fractals were presented sequentially on the scene image within the neuron's receptive field (presentation time, 400 ms; interstimulus interval, 400 ms). Liquid reward (0.2 mL) was delivered 300 ms after the last object was presented. The reward occurrence was thus not associated with any object. Each object was presented at least seven times in one session.

### No-Scene Object-Value Task.

This task was used for the object-value learning in the pharmacological experiment. The procedure was similar with scene-based object-value task, but no background scene was used and the eight objects were divided into good and bad objects without switching (*SI Appendix*, Fig. S4*A*). After the monkeys fixated on the white-square fixation point for 600 to 1,000 ms, the fixation cue disappeared and two objects (one good and one bad) appeared simultaneously in same hemifield. A reward was given after the monkeys make a saccade to the stimulus and maintained the fixation for 200 ms. The half of the fractal objects was associated with a large reward (0.3 mL; i.e., good objects), whereas the other half were associated with small reward (0.1 mL; i.e., bad objects). The reward outcome remained unchanged for each object.

### Recording Procedure.

Based on a stereotaxic atlas, a recording chamber was placed over the parietal cortex, tilted laterally by 25° (monkey WK) or 0° (monkey SP) and aimed at the striatum tail. MR images (4.7 T; Bruker) were then obtained along the direction of the recording chamber that was visualized by filling a recording grid with gadolinium.

To record from single neurons, a tungsten electrode (Alpha Omega Engineering or FHC) was lowered into the striatum through a guide tube using a micromanipulator (MO-97S; Narishige). The recording site was determined using a grid system, which allowed electrode penetrations at every 1 mm. We amplified and filtered (0.3 to 10 kHz; Model 1800, A-M Systems; Model MDA-4I, BAK) signals obtained from the electrodes and collected at 1 kHz. Single neurons were isolated online using the custom voltage–time window discriminator software (Blip).

### Pharmacological Manipulations.

To temporarily block inputs to FSI, the drug IEM-1460 (an inhibitor of GluA2-lacking AMPARs) was locally injected in the STRt and cdPUT. This drug selectively blocked synaptic excitation of FSIs but not MSNs or cholinergic interneurons (28, 31, 32). We first recorded the neuronal activity of FSIs and then injected IEM-1460. For this purpose, we manufactured injectrodes composed of epoxy-coated tungsten microelectrode (FHC) and silica tube (Polymicro Technology). The injectrode connected to a 10-μL Hamilton microsyringe and was inserted through the guide tube. A small amount of IEM-1460 (2.5 mM, 1 μL for each site) was pressure-injected using a manual infusion pump (Stoelting). The inactivation effects were assessed by comparing object choice before and 15 to 120 min after drug injection. We also injected saline in separate experiments to ensure that the effect was not due to any volume effect.

### Identification of MSNs and FSIs.

Using waveform and firing-rate criteria (27–29, 39, 40), we characterized the electrophysiological properties of recorded neurons (*SI Appendix*, Fig. S8). Baseline firing rate was computed as the mean firing rate during the 250 ms before the onset of the scene. We aligned the voltage traces of all recorded action potentials of each neuron by their peaks and calculated the median waveform of the action potentials. The median waveform was interpolated using a cubic spline function (Matlab). Then, the action potential duration of each neuron was defined as the time from the peak to the trough of the waveform. The classification was performed separately based on the amplifier that we used (BAK or AM system), since spike shapes are strongly dependent on amplifier filter settings or type (62, 63). Putative FSIs exhibited a peak–trough distance < 800 μs (or 480 μs) and a baseline firing rate > 2 Hz. Putative MSNs exhibited a peak–trough distance > 800 μs (or 480 μs) and a baseline firing rate < 10 Hz. We did not include the data from presumably tonically active neurons, which exhibited characteristic tonic firing pattern and wider action potentials (64). Units not meeting either of these criteria were excluded from the analysis (*SI Appendix*, Fig. S8).

### Data Analysis.

We defined the PUTt as the region 0 to 3.5 mm from the ventral edge of putamen, and the cdPUT as the region above it (11). Because the PUTt and CDt share the same anatomical pathway (44, 65) and showed the similar long-term value coding in the previous study (11), we combined neurons in these areas as STRt neurons. The off-line analyses were performed using Matlab (MathWorks). We defined object-responsive neurons as units that showed a significant response to any of eight or two fractal objects (50 to 400 ms after object onset) compared with the preobject period (250 ms before object onset; two-sided paired *t* test, *P* < 0.05 with Bonferroni correction). Similarly, we defined scene-responsive neurons as neurons that showed a significant response to either of two scenes (50 to 400 ms after scene onset) compared with the baseline period (250 ms before scene onset; two-sided paired *t* test, *P* < 0.05 with Bonferroni correction). We also defined the “preferred scene” based on the difference in the averaged responses to all objects for analyze the object-responsive neurons (Figs. 5*E* and 6*C*) and based on the difference in response to the two scenes for analyzing the scene-responsive neurons (Fig. 6*B* and *SI Appendix*, Fig. S10). The time course of neuronal activity for each condition was shown after smoothing with a Gaussian kernel (σ = 15 ms). We used only the data in correct trials for behavioral and neuronal analysis.

### Computational Model.

We adapted an established network model of value learning (66–68) and extended it to construct the model diagrammed in Fig. 8. In this model, MSN receives signals from two different inputs: The cerebral cortex and FSI. The cerebral cortex conveys object and scene information and makes the excitatory connections with MSNs (33). Synaptic plasticity of these connections is mediated by DA, which encodes the reward value (12–14). FSI also receives scene information from the cortex and makes inhibitory connections onto MSN. Based on this neuronal circuit, the response of MSN is defined as

$$\text{MSN}=\left(I_{OA}\times S_{OA}+I_{OB}\times S_{OB}+I_{SX}\times S_{SX}+I_{SY}\times S_{SY}\right)÷\left(I_{SX}\times FSI_{SX}+I_{SY}\times FSI_{SY}\right)$$

where *I*_*OA*_, *I*_*OB*_, *I*_*SX*_, and *I*_*SY*_ denote whether object A, object B, scene X, and scene Y were presented (1) or not (0), respectively. *S*_*OA*_, *S*_*OB*_, *S*_*SX*_, and *S*_*SY*_ denote the synaptic weight of cortical inputs for object A, object B, scene X, and scene Y, respectively. *FSI*_*SX*_ and *FSI*_SY_ denote inhibitory inputs from FSIs for scenes X and Y, respectively. Changes in synaptic weight are implemented as follows:

$$S_{OA}=S_{OA}+I_{OA}\times MSN\times \mathrm{Reward}\times \mathrm{LS}$$

$$S_{OB}=S_{OB}+I_{OB}\times MSN\times \mathrm{Reward}\times \mathrm{LS}$$

$$S_{SX}=S_{SX}+I_{SX}\times MSN\times \mathrm{Reward}\times \mathrm{LS}$$

$$S_{SY}=S_{SY}+I_{SY}\times MSN\times \mathrm{Reward}\times \mathrm{LS}$$

where *Reward* denotes whether reward was presented (1) or not (−0.5), and *LS* denotes the coefficient of the learning speed for cortical inputs for objects and scenes. We set the values of *FSI*_*SX*_ and *FSI*_*SY*_ as 0.6 and 1 (for normal condition) (*SI Appendix*, Fig. S12*A*) or 0.5 and 0.5 (for low FSI input condition) (*SI Appendix*, Fig. S12*B*), respectively. We set the value of *LS* as 0.01 and the initial values of *S*_*OA*_, *S*_*OB*_, *S*_*SX*_, and *S*_*SY*_ as 1.

### Histological Procedures.

At the end of the experiments, several electrolytic lesions were made at or near the sites where the task-related neurons were recorded. Lesions were made by passing a direct current through the recording electrodes (13 μA, tip negative) for 20 s. The monkey was deeply anesthetized with an overdose of sodium pentobarbital (390 mg/mL) and perfused transcardially with saline followed by 4% paraformaldehyde. The head was fixed in a stereotaxic frame, and the brain was cut into blocks in the coronal plane including the midbrain region. The block was postfixed overnight at 4 °C, and then cryoprotected for 1 wk in increasing gradients of glycerol solution (5%, 10% to 20% glycerol in PBS) before being frozen. The frozen block was cut every 50 μm using a microtome. Slices taken every 250-μm interval were taken and used for Nissl staining and photomicrographs. The locations of task-related neurons were reconstructed according to the depth and the coordinates of electrode penetrations and the relative locations of the marking lesions.

### Immunohistochemistry.

To examine the distribution of FSIs in the STRt, we stained the PV by immunohistochemistry and checked the location of them. The sections were preincubated for 30 min in 0.3% hydrogen peroxide in 0.1 M PBS (pH 7.2) to block endogenous peroxide, followed by three rinses through 0.1 M PBS, and then 1 h in blocking solution containing 0.03% Triton X-100, 0.2% BSA, and 3% normal horse serum in 0.1 M PBS. The sections were incubated in blocking solution with primary antibody (monoclonal mouse anti-PV antibody, 1:2,000 dilution, Sigma-Aldrich) overnight at room temperature. The sections were rinsed three times with PBS, then incubated for 60 min with a biotinylated secondary antibody (biotinylated horse anti-mouse antibody, 1:400 dilution; Vector Laboratories). After another series of PBS rinses, sections were incubated for 90 min in avidin–biotin–peroxidase complex before the visualization step, for which we used a Vector NovaRed kit (Vector Laboratories). After staining the sections were mounted on glass slides, dehydrated in ethanol, cleared in xylene, and cover-slipped. We identified the stained neurons from four slides (AC-6 and AC-7 in each monkey; 6- or 7-mm posterior to the anterior commissure) using a microscope (Keyence) and a confocal laser scanning microscope (Zeiss). To quantify the distribution of FSIs, we measured the density of labeled cells in each region (CDt, cvPUT, and cdPUT) and normalized by the highest labeled cell density in these areas (Fig. 2*A*).

## Supplementary Material

Supplementary File

## Data Availability

The mat file data have been deposited as “Environment-based object values learned by local network in the striatum tail” at https://data.mendeley.com/datasets/t9gp59g8p5/draft?a=2a537090-8765-461d-8484-7c99e060bd44).
