# Supplementary material for: Environment-based object values learned by local network in the striatum tail
Source: Proc Natl Acad Sci U S A. 2021 Jan 18;118(4):e2013623118. doi: 10.1073/pnas.2013623118 (PMC7848585; doi:10.1073/pnas.2013623118)
Supplement: Supplementary File [file pnas.2013623118.sapp.pdf]

PNAS
www.pnas.org

Supplementary Information for

Environment-based object values learned by local network in the striatum tail

Jun Kunimatsu, Shinya Yamamoto, Kazutaka Maeda and Okihide Hikosaka

* Correspondence: Jun Kunimatsu
Email: kunimatsu.jun@gmail.com

This PDF file includes:

SI Materials and Methods
Figures S1 to S12
SI References

SI Materials and Methods

Object Value Coding (Scene Absent)

To examine the long-term effect object reward value learning, we used the stable value procedure (No-scene object value task) (1, 2). This procedure contained no scene and eight objects that were divided into good and bad objects without switching (Fig. S4*A*). Monkeys learned the object value by making a saccade to the one of eight objects and get the associated reward (good, large-reward, 0.3 ml; bad, small-reward, 0.1 ml). The same sets of fractals were used repeatedly for learning for 5 days, throughout which every object retained a consistent value (either good or bad). The passive-viewing task was used to test neurons for long-term stable value coding. This task was same as the task used in scene-based value condition (Fig. S1*B*), except that the scene was not presented.

Free Viewing Task

This task was used to examine how the monkey responded to valued objects depending on the scene, but without any reward outcome (Fig. S1). After the monkey fixated on a central white dot for 300 ms, four objects were presented simultaneously in four symmetric positions (15° from center) on the scene background. In any given trial, four fractals and the scene were randomly chosen from a scene-object set. The monkey was free to look at them for 2000 ms without any reward outcome. After a blank period (500-700 ms), a white dot was presented at one of eight positions. If the monkey made a saccade to the dot a liquid reward was delivered (0.2 ml). Each object was presented on each scene image at least 10 times in one session.

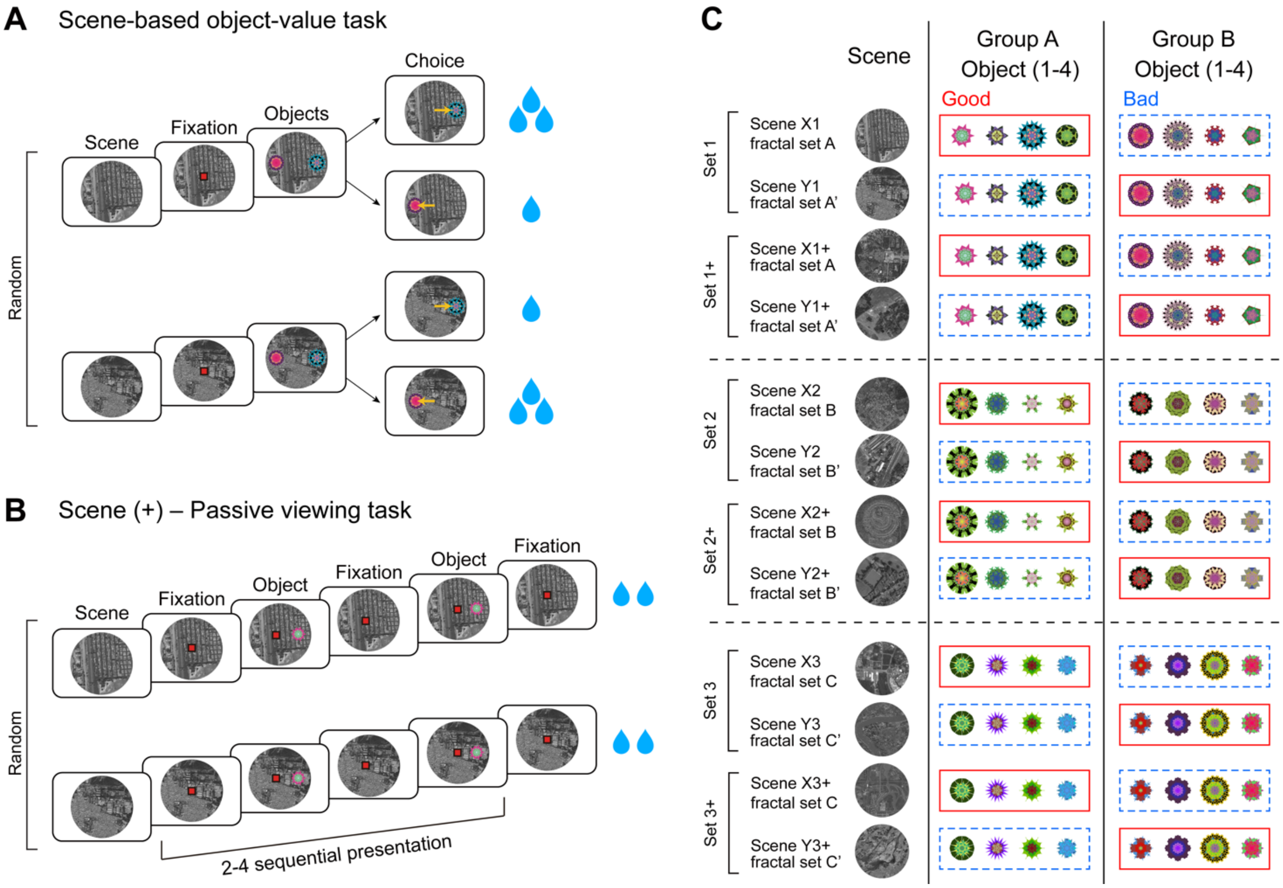

Fig. S1 (related to Fig. 1). Learning and testing procedure for the scene-based object values
(*A*) Learning: Scene-based object-value task. After an inter-trial interval, one of two scenes appeared pseudo-randomly, followed by the appearance of the fixation point (red square). After the monkey's fixation two objects with different values (good and bad) appeared simultaneously as the fixation point disappeared. The monkey was required to make a saccade to one of the targets and fixate on it to get the outcome associated with that object. The amount of reward was reversed when the scene changed for each object. When 8 objects were used as shown in (*C*), the two objects were chosen pseudo-randomly, 1 from group A and 1 from group B. (*B*) Testing: Scene(+)-passive viewing task. The starting procedure is the same as learning (*A*), but the monkey needed to keep fixating gaze at the center while a random number of objects (2-4), which were chosen pseudo-randomly from a set of 8 objects (see *C*), were presented serially in the neuron's receptive field, followed by a fixed amount of reward. Thus, objects were not associated with the consistent reward during this test. (*C*) Multiple sets of scenes and objects. Each experimental set contained 2 scenes and 8 fractal objects. One of these sets was chosen in each experiment. Each set of 8 objects was tested in 2 sets of scenes (e.g., Set 1 and Set 1+). In total, 12 scenes and 24 objects were used.

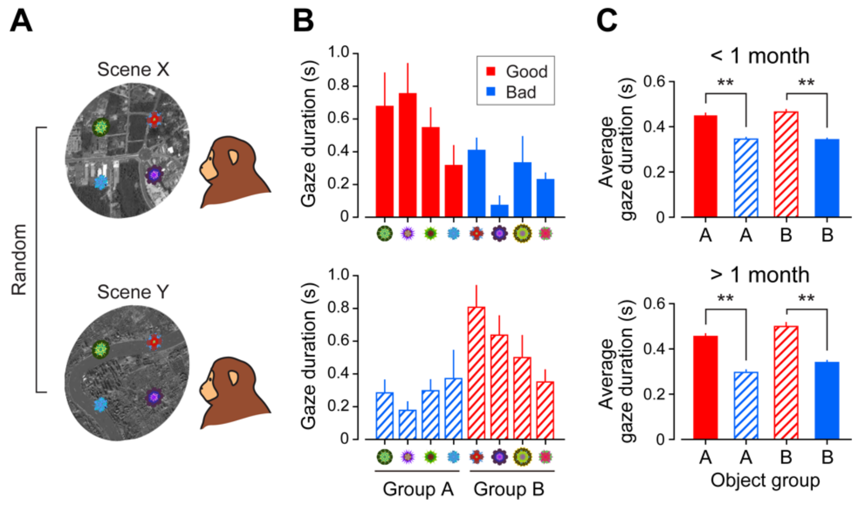

Fig. S2 (related to Fig. 1). Gaze bias after scene-based object-value learning
(*A*) Free viewing task. 4 fractal objects appeared simultaneously in one scene and the monkey was free to look at them for 2 s. On each trial the scene and 4 objects were chosen pseudorandomly from 1 scene-fractal set (Fig. S1C). (*B*) Example data (monkey WK): gaze durations on a set of 8 fractal objects in scene X (*top*, filled) and scene Y (*bottom*, shaded) during one session of the free-viewing task. (*C*) The average of gaze duration on good or bad objects that within 1 month (*top*, n = 58, n = 31 in monkey WK, n = 27 in monkey SP) and over 1 month (n = 26, n = 19 in monkey WK, n = 7 in monkey SP) after learning. Significant value-coding is indicated by asterisk (**P < 0.01, two-sided Wilcoxon rank-sum test). Note that the monkeys switched their object choices automatically according to the scene.

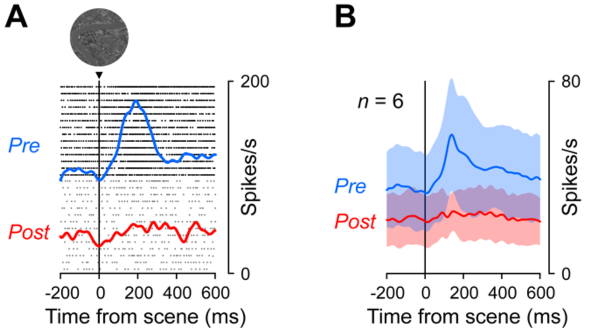

Fig. S3 (related to Fig. 3). Effect of IEM-1460 on FSIs.
(*A*) Response of an example FSI to a scene before (Pre) and after (Post) the local injection of IEM-1460 around the FSI. Its spontaneous firing rate was also reduced. (*B*) Averaged responses of 6 FSIs before (Pre) and after (Post) IEM-1460 injection.

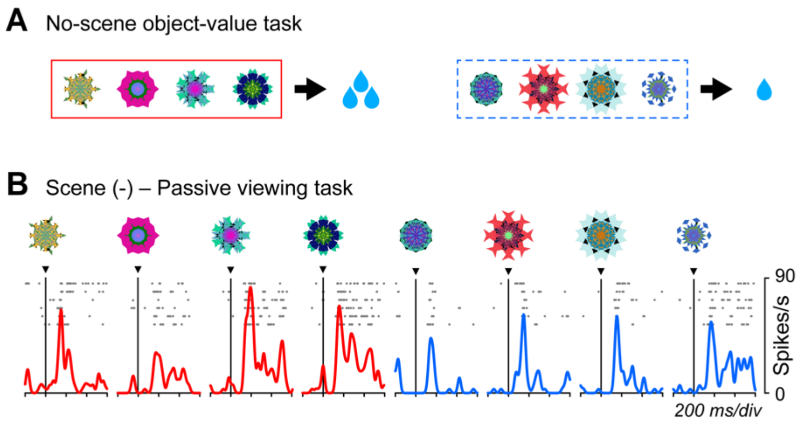

Fig. S4 (related to Fig. 4). No-scene object-value task

(*A*) Among a set of 8 fractal objects, 4 were associated with a large reward (called good objects) and the other 4 were associated with a small reward (called bad objects). This learning was done with no change of object values across several days. (*B*) Responses of a MSN in STRt to the learned objects during the scene(-)-passive-viewing task. This task was done 14 days after the last learning. Data are aligned on the onset of each object. The MSN responded to good objects more strongly than to bad objects, although it also showed object-selective responses. This feature is common to MSNs in STRt, but their outputs together show clear value-coding while object-selectivity becomes weaker, which is shown in the downstream area including SNr (1, 3).

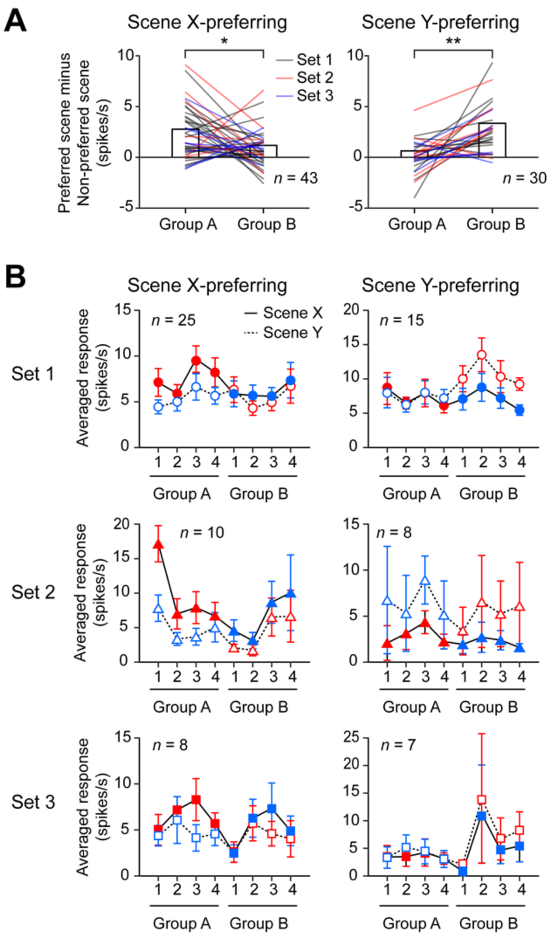

Fig. S5 (related to Fig. 4 and 5). Responses to the objects in each preferred scene

(*A*) Response changes of individual MSNs to the same objects across scenes (equivalent to the population data in Fig. 5*B* and *D*). Responses of scene X-preferring MSNs (*left*) and scene Y-preferring MSNs (*right*) to group-A objects and group-B objects (see Fig. S1*C*). Among scene X-preferring MSNs (*left*), the response tended to be larger to group A (Good) objects than group B (Bad) objects in scene X. Among scene Y-preferring MSNs (*right*), the response tended to be larger to group B (Good) objects than group A (Bad) objects in scene Y. Different scene-object sets (Fig. S1*C*) are shown by different colors. Outlier points has been omitted from each of the plots in *left* ([18.1, 13.7]) and *right* panels ([4.8, 12.6], [12.0, 11.8]) but are included in the bar graph. (*B*) Response of MSNs to individual objects, shown separately for scene X (solid line) and scene Y (dotted line). Data are shown separately for 3 groups of objects, each tested with 4 scenes (Fig. S1*C*). The number of MSNs examined is shown in each graph. Object values are indicated by color (red: good objects, blue: bad objects). Note that MSN responses to good objects tended to be stronger in the preferred scene.

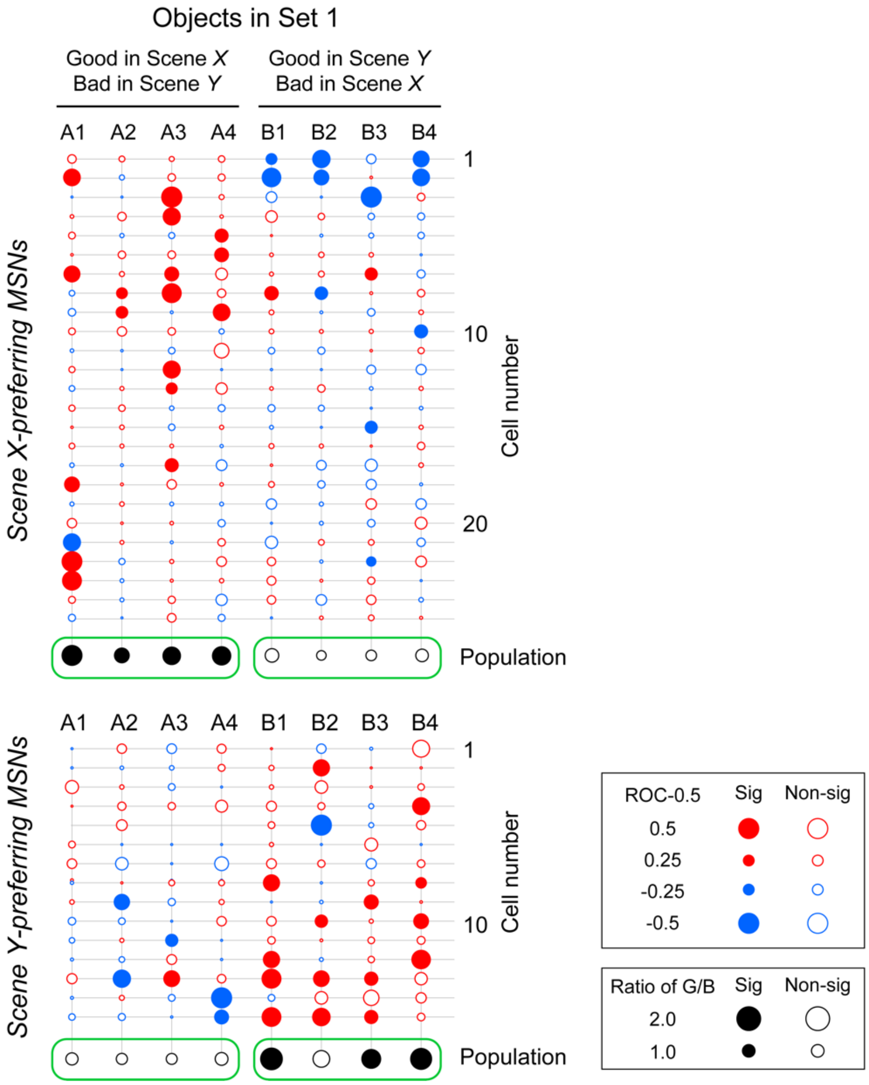

Fig. S6 (related to Fig. 4 and 5). Value coding of individual MSNs for each object

Responses to objects in set 1 (Fig. S1) are shown for scene X-preferring MSNs (*top*, *n* = 25) and scene Y-preferring MSNs (*bottom*, *n* = 15). Each MSN's responses to 8 objects (A1-4, B1-4) are shown in a horizontal line. Circle color means the direction of value: Red = positive value (good > bad), Blue = negative value (bad > good). Circle size indicates value modulation index (ROC-0.5). Filled circle indicates statistically significant value (Wilcoxon ranksum test, $P < 0.05$). At the bottom of each group of MSNs is shown the value-coding of each object by these MSNs together (Population): statistically significant (black) and non-significant (white). Circle size indicates the ratio of response (good/bad). These Population data indicate that MSNs in each group, together, generate value-coding for each object (mostly) if it was good in their preferred scene: Object A1-4 (*top*) and Object B1, 3, 4 (*bottom*). These data correspond to Fig. 5 *B* and *D*.

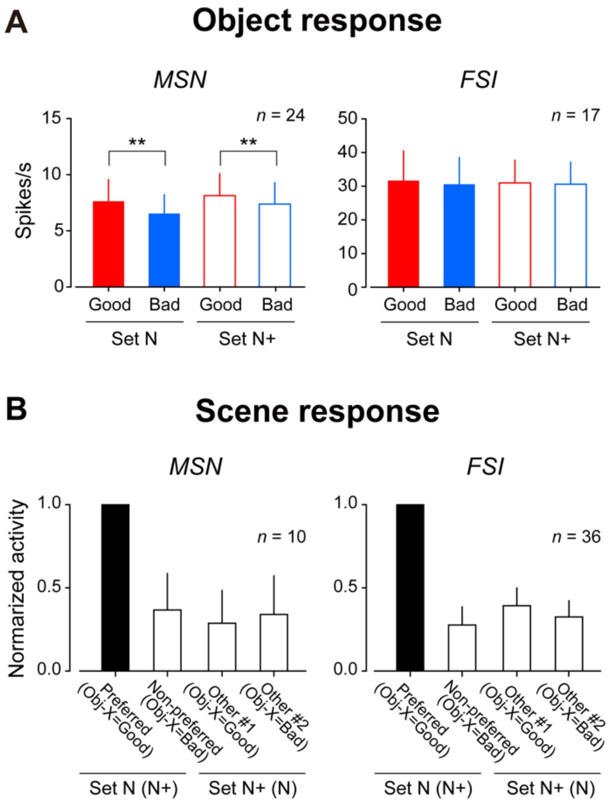

Fig. S7 (related to Fig. 4 and 5). Responses to multiple objects and scenes

(*A*) Average responses of MSNs (*left*) and FSIs (*right*) to good objects (e.g., object A in scene X and object B in scene Y) and bad objects (e.g., object A in scene Y and object B in scene X), which were tested with two sets of scenes: Set N (e.g., Set 1 in Fig. S1*C*) and Set N+ (e.g., Set 1+ in Fig. S1*C*). MSNs discriminated many objects (*n* = 24) by their values, even though their values were switched across many scenes (*n* = 12) (*left*). In contrast, FSIs did not discriminate the many objects by their values, regardless of the scenes (*right*). (*B*) Variable responses of individual neurons to the two sets of scenes (e.g., Set 1 and 1+ in Fig. S1*C*) in MSNs (*left*) and FSIs (*right*). The scene that the neuron showed the strongest response was defined as "Preferred". "Non-preferred" is another scene in the set with "Preferred". "Other #1" and "Other #2" are the scenes in the different set with "Preferred". "Other #1" and "Other #2" are the scenes that have the same and different value of objects as "Preferred", respectively.

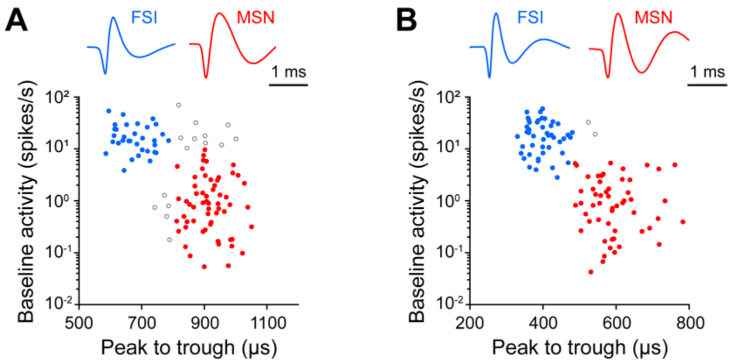

Figure S8 (related to Fig. 6). The action potential duration and baseline firing of all neurons
(*A*) Classification of neurons which recorded by Bessel filter (BAK) as MSNs (red) or FSIs (blue) by spike width (abscissa) and firing rate (ordinate). Unclassified neurons (gray) were plotted by unfilled dots. Inset shows mean waveforms of each type of neurons. Inset shows mean waveforms of each type of neurons. (*B*) Neurons recorded by Butterworth filter (A-M system).

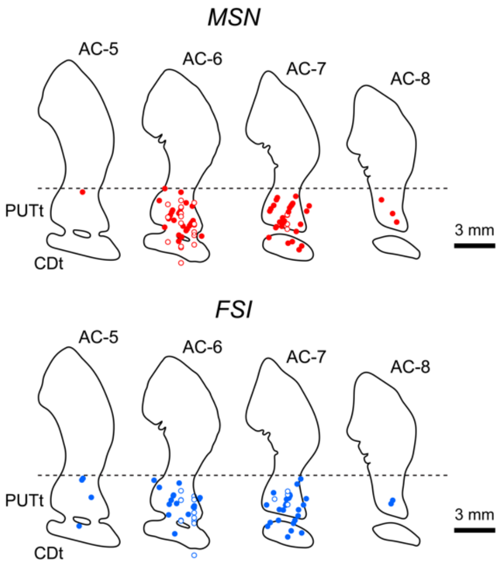

Fig. S9 (related to Fig. 6). Location of recorded neurons
Locations of visual neurons in STRt (PUTt and CDt) that responded to scenes and/or objects: MSNs (*top*) and FSIs (*bottom*). Horizontal dashed line indicates the dorsal boundary of STRt, shown in coronal sections 5-8 mm posterior to the anterior commissure (2). PUTt and CDt are separated by a thin white matter layer. Filled and Unfilled dots indicate the neurons in monkey WK and monkey SP, respectively.

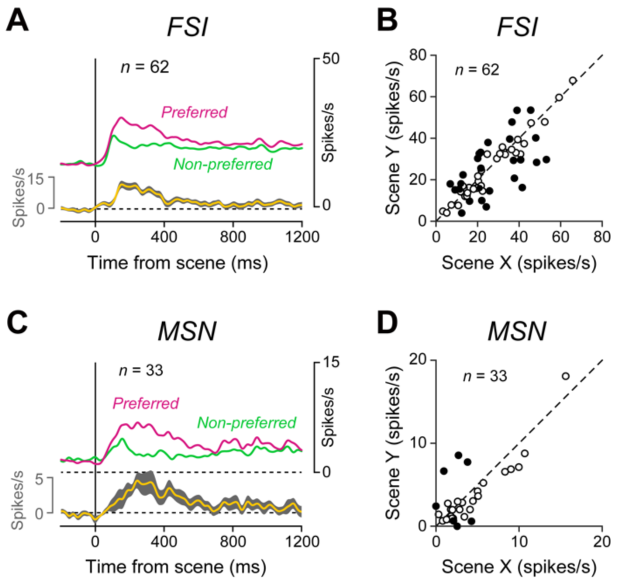

Fig. S10 (related to Fig. 6). Response of FSIs and MSNs to scene

(*A*) Time course of averaged responses of FSIs to the preferred scene (magenta) and the non-preferred scene (green). This figure is completely the same as Fig. 6*B*. (*B*) Each data point compares the response of MSNs to scene X and scene Y. Filled symbols indicate the data showing a significant difference (Wilcoxon ranksum test, $P < 0.05$). (*C* and *D*) Responses of MSNs in the same form as in (*A* and *B*).

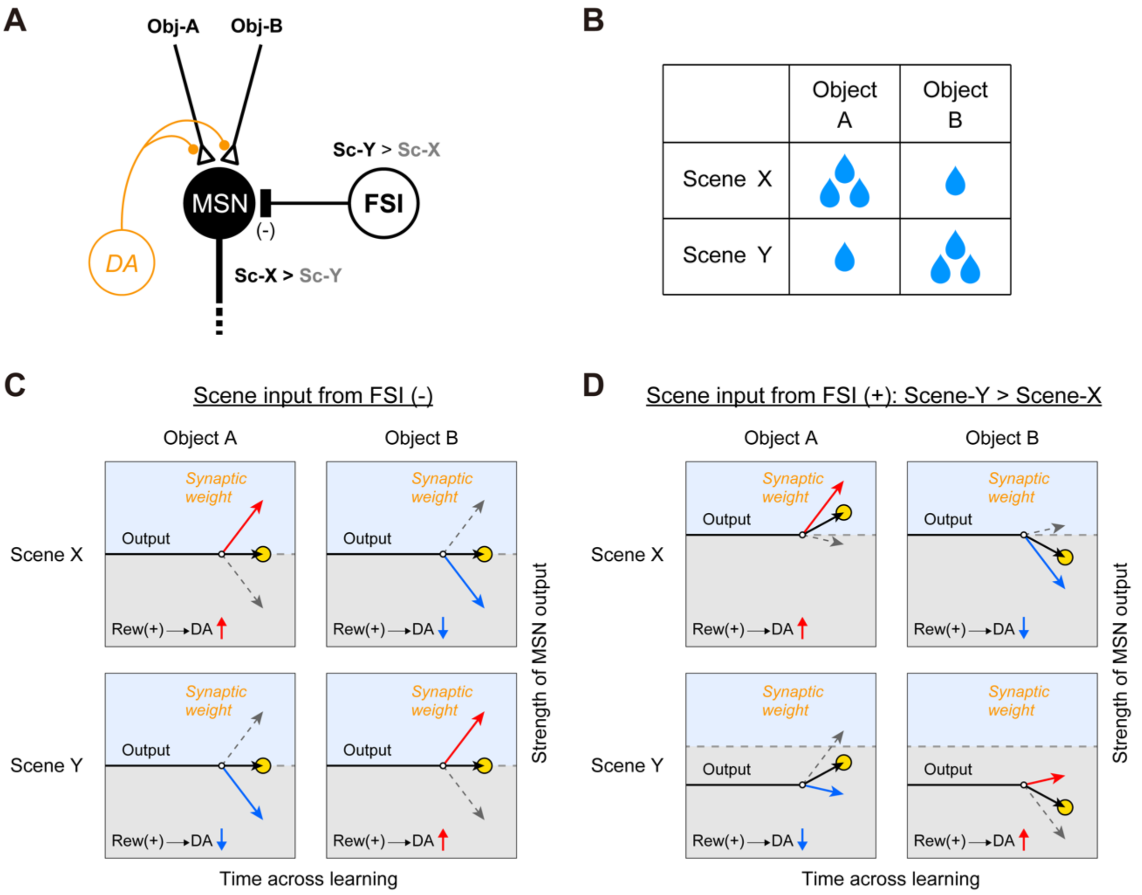

Fig. S11 (related to Fig. 8). Hypothetical mechanism of scene-based object-value learning
(*A*) Striatal local circuits receiving selective object information and reward-related dopamine input. If object A leads to a reward, dopamine (DA) neurons are excited and gradually increases the synaptic weight of object A input to MSN. If no reward, DA neurons are inhibited and decrease the synaptic weight of object A input. These effects are modulated by the inhibitory input from FSI which is selective to scenes (Fig. 6), as shown in D. (*B*) Scene-based object-value task (Fig. 1*B*) which was learned by monkey subjects. The value of object A is high (good) in scene X, but it is low (bad) in scene Y. The value of object B is low (bad) in scene X, but it is high (good) in scene Y. (*C*) Changes in synaptic weight of object inputs to MSN in the 4 conditions without scene input from FSI. The synaptic weight of object A input increases in scene X (due to a reward) (shown by red arrow in *top-left*), but decreases in scene Y (due to no reward) (shown by blue arrow in *bottom-left*). Since these conditions occur randomly across trials and the changes in the synaptic weight occur gradually, the object A input to MSN will rarely change (shown by black arrow with yellow circle in both *left-up* and *left-down*). The same effect would occur for object B input (*top-right* and *bottom-right*). (*D*) Changes in synaptic weight of object inputs to MSN in the 4 conditions with scene input from FSI. If FSI is more excited by scene Y than scene X (as shown in A), the output of MSN decreases in scene Y than scene X (*bottom*). Then, the effect of DA input on the change of the synaptic weight is reduced, for either increase (*bottom-right* compared with *top-left*, shown by red arrow) or decrease (*bottom-left* compared with *top-right*, shown by blue arrow). These effects together lead to the selective response of objects based on the scene (shown by black arrow with yellow circle in each condition): stronger response to object A in scene X (*top-left*). Other responses would be weaker: object A in scene Y, object B in scene X, object B in scene Y. If MSN receives FSI input which is stronger to scene X than scene Y, the MSN would respond differently: stronger response to object B in scene Y (*bottom-right*). See Figure 8.

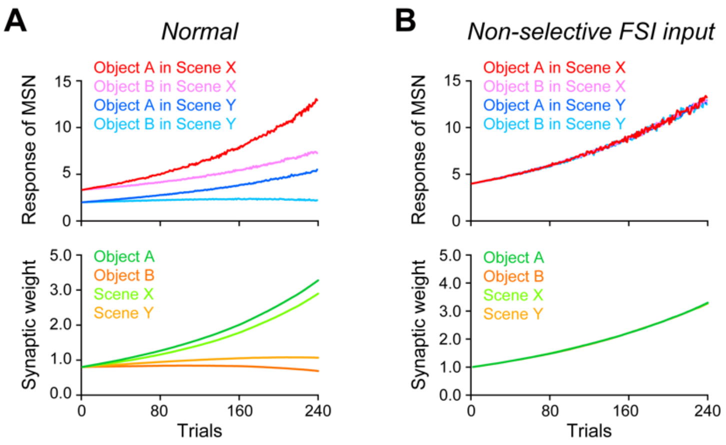

Fig. S12 (related to Fig. 8). Computational model data of scene-based object-value learning.
The computation model is shown in Figure 8 and explained in Figure S11. (*A*) Changes in the activity of scene-X preferring MSN during learning in the normal condition, which was calculated by the computational model (average of 1000 simulated data): Responses to two groups of objects (*A and B*) changed differently in two scenes (X and Y) (*top*) (see Fig. S11*D*). This is based on the changes in the synaptic weights of individual inputs (objects and scenes) (*bottom*) (see Fig. 8, *left*). Note that the response of MSN is different depending on the scene-object combination. (*B*) Changes in the activity of MSN during learning when FSI's inhibitory input is blocked. Then, MSN's response to any excitatory input (object, scene) would not become discriminative, since the outcome of any of the inputs is reward (+) or reward (-) equally, based on the two scenes (see Fig. S11*C*).

SI References

1. M. Yasuda, S. Yamamoto, O. Hikosaka, Robust representation of stable object values in the oculomotor Basal Ganglia. *J. Neurosci.* **32**, 16917-16932 (2012).
2. J. Kunimatsu, K. Maeda, O. Hikosaka, The caudal part of putamen represents the historical object value information. *J. Neurosci.* **39**, 1709-1719 (2019).
3. M. Yasuda, O. Hikosaka, Functional territories in primate substantia nigra pars reticulata separately signaling stable and flexible values. *J. Neurophysiol.* **113**, 1681-1696 (2015).
